# Identification of RECK as a protective prognostic indicator and a tumor suppressor through regulation of the ERK/MAPK signaling pathway in gastric cancer

**DOI:** 10.1186/s12967-023-04644-z

**Published:** 2023-10-30

**Authors:** Fangyuan Qi, Yaru Wang, Bingxin Yu, Fan Li

**Affiliations:** 1 The Key Laboratory of Zoonosis, Department of Pathogenobiology, Chinese Ministry of Education, College of Basic Medicine, Jilin University, No. 126 Xinmin Street, Changchun, 130021 Jilin People’s Republic of China; 2 Department of Ultrasound, The Third Hospital of Jilin University, Changchun, Jilin People’s Republic of China; 3 The Key Laboratory for Bionics Engineering, Ministry of Education, Jilin University, Changchun, People’s Republic of China; 4 Engineering Research Center for Medical Biomaterials of Jilin Province, Jilin University, Changchun, People’s Republic of China; 5 Key Laboratory for Health Biomedical Materials of Jilin Province, Jilin University, Changchun, People’s Republic of China; 6 State Key Laboratory of Pathogenesis, Prevention, and Treatment of High Incidence Diseases in Central Asia, Urumqi, Xinjiang People’s Republic of China

**Keywords:** Gastric cancer, RECK, Tumor suppressor, ERK/MAPK, CALD1

## Abstract

**Background:**

Gastric cancer (GC) ranks as the fifth most common cancer worldwide and is characterized by its significant heterogeneity and unfavorable prognosis. Thus, identifying efficient prognostic factors and understanding the underlying molecular mechanisms in GC are essential for improving patient outcomes. In this study, we aimed to investigate the role of RECK (reversion-inducing cysteine-rich protein with Kazal motifs) in the prognostic significance and molecular mechanisms of its biological function in GC.

**Methods:**

Multiple bioinformatics strategies were performed to detect the potential functions and prognostic efficiency of RECK in GC. Rescue experiments revealed that the molecular mechanism by which RECK in inhibited tumor proliferation, migration, and invasion was mediated by ERK/MAPK signaling in AGS and HGC-27 cells. Using integrated bioinformatics analysis and western blot assay, we investigated the potential interaction between CALD1 and RECK.

**Results:**

Our findings revealed significantly decreased RECK expression in GC samples compared to normal samples and RECK was identified as a promising predictor for the prognosis of GC patients. Moreover, upregulation of RECK demonstrated a distinctly positive association with a high-immunity and low-metastasis microenvironment in GC. Mechanistically, the antitumour effects of RECK on hampering tumor cell growth, migration, and invasion were mediated by the ERK/MAPK signaling pathway. In addition, we also illustrated that RECK inhibited the phosphorylation of CALD1 mediated by decreased phosphorylation of ERK.

**Conclusions:**

RECK is a promising prognostic biomarker and may shape a high-tumor-immunity and low-metastasis microenvironment in patients with GC. Moreover, RECK exerted its tumor-suppressive effects by the inactivation of ERK/MAPK signaling in GC cells.

**Supplementary Information:**

The online version contains supplementary material available at 10.1186/s12967-023-04644-z.

## Introduction

Gastric cancer (GC) is the fifth most commonly diagnosed cancer with an estimated 1.1 million new cases and the fourth leading cause of cancer-related death with an estimated 0.77 million deaths worldwide in 2020 [1]. Most GC patients are frequently diagnosed with an advanced stage and metastasis, resulting in a dismal prognosis with a 5-year overall survival rate of less than 30% [2]. It is imperative to improve the overall survival rate for GC patients through early diagnosis and effective targeted therapy. Consequently, there is an urgent need to identify specific and sensitive prognostic molecular markers for early diagnosis and new treatments and elucidate the molecular mechanism underlying the tumorigenesis and metastasis of GC. Given the challenging prognosis faced by many GC patients, this study seeks to explore the potential of RECK as a prognostic molecular marker and elucidate its underlying mechanisms in tumorigenesis and metastasis.

Reversion-inducing cysteine-rich protein with Kazal motifs (RECK), a negative cell surface regulator of matrix metalloproteinases (MMPs), is strongly suppressed in the majority of malignant tumors and functions as a suppressor of malignant tumor behaviors, including cell proliferation, invasion and metastasis [3–7]. Upregulation of RECK has been reported to be positively associated with immunogenic and hypovascularity status in hepatocellular carcinoma, suggesting that RECK expression may reflect a connection between immunogenic and hypovascularity [8]. Moreover, RECK suppresses cancer stem cells (CSCs) self-renewal and maintenance via mediating the activation of Notch1 signaling regulated by miR-221/22 in non-small cell lung cancer [9]. These reports indicate that RECK potentially affects tumor immunity status and CSCs gene expression. Nevertheless, there is limited knowledge of the potential molecular mechanisms for the antitumour effects of RECK in GC.

In the current study, bioinformatics analysis of diverse datasets revealed an aberrant decrease in RECK expression and an antitumour role for RECK as an independent factor contributing to favorable prognosis in patients with gastric cancer. Meanwhile, mechanistic investigations demonstrated that RECK remarkably suppressed tumor growth and the EMT process mediated by the inactivation of ERK/MAPK signaling.

## Methods

### Data collection and processing

The RNA and miRNA transcriptome data and clinical characteristics of stomach adenocarcinoma (STAD), colon adenocarcinoma (COAD), esophageal carcinoma (ESCA), and rectum adenocarcinoma (READ) were downloaded from The Cancer Genome Atlas (TCGA) database. The immune subtypes and stemness scores of cancers were downloaded from the UCSC Xena database. The GSE26899, GSE66229, GSE84437, GSE13681, GSE54129, GSE15459, GSE34942, and GSE26901 datasets for GC were retrieved from the Gene Expression Omnibus (GEO) database. The protein levels of RECK in pan-cancer datasets was obtained from the CPTAC database. The R package “edgeR” was employed for the TCGA count value, whereas the “limma” package was used to analyze chip data. FDR < 0.05 with |log2FC| ≥ 1 were considered the screening criteria for differentially expressed genes (DEGs). The expression data of normal gastric tissues were downloaded from the Genotype-Tissue Expression (GTEx) platform. Eighteen genes were obtained to calculate the T-cell inflamed score and 22 inhibitory immune checkpoints reported previously were obtained [10, 11]. Gene sets associated with the epithelial-to-mesenchymal transition (EMT) process were retrieved from the EMTome platform [12].

### Function and pathway enrichment analyses

Gene Ontology (GO), Kyoto Encyclopedia of Genes and Genomes (KEGG), Gene Set Enrichment Analysis (GSEA), and gene set variation analysis (GSVA) enrichment analyses were conducted by using the R package “clusterProfiler” with *P* < 0.05. Single sample Gene Set Enrichment Analysis (ssGSEA) were conducted by using the R package “GSVA” and “GSEABase”.

### Tumor microenvironment estimation (TME)

The R package “ESTIMATE” was applied to estimate the immune, stromal, and ESTIMATE scores. The relative fractions of TICs in STAD samples were obtained from the TIMER2.0 platform. The activities of the cancer immunity cycle were calculated using the TCGA RNA-seq count value on the TIP platform [13].

### Cell culture and transfection

GES-1, AGS, and HGC-27 cells, obtained from the Shanghai Cell Bank of Chinese Academy of Medical Sciences (Shanghai, China), were cultured in high glucose Dulbecco’s Modified Eagle’s media (DMEM; HyClone, Logan, Utah, USA) supplemented with 10% (v/v) fetal bovine serum (FBS; Gibco, Grand Island, NY, USA) and 1% penicillin-streptomycin (MRC, Jintan, China) at 37 °C under a 5% CO_2_ atmosphere. The coding sequence of human RECK or CALD1 was cloned into the pcDN3.1 vector for overexpression (PPL, Nanjing, China). The sequences of three siRNAs targeting RECK for knockdown are shown in Additional file 1: Table S1 (RIBOBIO, Guangzhou, China). Three siRNAs targeting CALD1 for knockdown were purchased from GenePharma company (GenePharma, Suzhou, China). X-treme GENE HP DNA Transfection Reagent (Roche, Shanghai, China) was used for cell transfection following the manufacturer’s protocol. After transfection for 24 h, GC cells were treated with 10 µM PD98059 (an ERK inhibitor) or 10 µM *tert*-butylhydroquinone (TBHQ, an ERK activator) (MedChemExpress, America) and then cultured for 24 h for subsequent research.

### Reverse transcription quantitative real-time polymerase chain reaction (RT-qPCR)

Total RNA was extracted using the RNAsimple Total RNA Kit (TIANGEN, Beijing, China). Reverse transcription was performed with the HiScript II First Strand cDNA Synthesis Kit (Vazyme, Nanjing, China) following the manufacturer’s protocols. Then, real-time PCR was conducted using FastStart Universal SYBR Green Master Mix (ROX) (Roche, Switzerland) on an ABI PRISM 7300. The primer sequences used are listed in Additional file 1: Table S2.

### Western blotting

After extracting the total protein with the Column Tissue & Cell Protein Extraction Kit (Yamei, Shanghai, China), the proteins were separated by 10% SDS-PAGE and then transferred onto polyvinylidene fluoride membranes (Thermo Fisher, Waltham, MA, USA). Next, the membranes were blocked with 5% skim milk for 2 h, incubated with primary antibodies at 4 °C overnight, including β-actin (Abcam, diluted 1:5000, ab6276), RECK (Cell Signalling Technology, diluted 1:1000, #3433S), CALD1 (Abcam, diluted 1:10,000, ab32330), p-CALD1 (Abcam, diluted 1:1500, ab76106, phospho S759), GAPDH (Cell Signalling Technology, diluted 1:1000, #5174), Histone (Proteintech, diluted 1:2000, #17168-1-AP), p21 (Cell Signalling Technology, diluted 1:1000, #10355-1-AP), PCNA (Cell Signalling Technology, diluted 1:20,000, #10205-2-AP), E cadherin (Cell Signalling Technology, diluted 1:1000, #14472), N cadherin (Cell Signalling Technology, diluted 1:1000, #4061S), Vimentin (Cell Signalling Technology, diluted 1:1000, #5741S), MMP2 (Cell Signalling Technology, diluted 1:1000, #87809S), p38 (Cell Signalling Technology, diluted 1:1000, #8690S), p-p38 (Cell Signalling Technology, diluted 1:1000, #4511S), JNK1/2 (Cell Signalling Technology, diluted 1:1000, #3708S), p-JNK1/2 (abcm, diluted 1:1000, ab4821), ERK (Cell Signalling Technology, diluted 1:1000, #4695S), p-ERK (Cell Signalling Technology, diluted 1:1,000, #4370S), MEK (Cell Signalling Technology, diluted 1:1000, #4694S), p-MEK (Cell Signalling Technology, diluted 1:1000, #9154S). Then the membranes were treated with a horseradish peroxidase-conjugated secondary antibody (Bioss, Beijing, China) for 1 h. Enhanced chemiluminescence reagents (Beyotime, Shanghai, China) were used to detect and capture images of the protein bands.

### Cell viability assays

AGS and HGC-27 cells were seeded into 96-well microplates at a density of 4000 cells per well. Cell growth was examined by the Cell Counting Kit 8 (CCK-8) assay (MedChemExpress, America) according to the manufacturer’s instructions. The absorbance at 450 nm was determined using a microplate spectrophotometer after the cells were cultured for 24, 48, and 72 h.

### EdU assay

AGS and HGC-27 cells were seeded into 6-well plates, and cell proliferation was tested by the EdU assay (Beyotime, Shanghai, China) according to the manufacturer’s instructions. Finally, fluorescence images of GC cells were captured under an Olympus fluorescence microscope.

### Colony formation assay

Briefly, AGS and HGC-27 cells were seeded into 6-well plates and cultured at 37 °C under 5% CO_2_. After 10 days of treatment, colonies were fixed with 4% paraformaldehyde and stained with Giemsa (Beyotime, Shanghai, China). The number of colonies was calculated and photographed.

### Wound healing cell migration assay

GC cells were seeded into a 6-well plate and cultured to 90% confluence for a scratch using a 200 µl pipette tip in the middle of each well. Then each well was washed with PBS to remove loose cell debris, and replenish the plate was replenished with fresh low-serum culture medium (2% FBS). After 24 h of incubation, images were captured by an Olympus microscope, and the cell migration rate was calculated using ImageJ software.

### Matrigel invasion assay

In the invasion assay, the 24-well transwell chamber was coated with Matrigel matrix (Corning, USA) following the manufacturer’s instructions. GC cells were seeded onto the upper chamber wells in serum-free culture medium and the lower chamber was supplemented with medium containing 20% FBS. After 24 h of incubation, the cells in the upper chamber were removed and the cells in the lower chamber were fixed and stained. The number of infiltrating cells was counted in three randomly selected microscopic fields of each chamber.

### Cell cycle analysis

GC cells were harvested and fixed with 70% ethyl alcohol overnight at 4 °C. Then, the cells were stained with 500 ml PI/RNase staining buffer (BD Pharmingen, USA) for 15 min at 37 °C and analysed by flow cytometry. FlowJo software was used for quantitative analysis of the cell cycle distribution.

### Cell apoptosis analysis

GC cells were collected and washed twice with PBS and then stained following the manufacturer’s instructions for the Annexin V-FITC Apoptosis Detection Kit (BD Pharmingen, USA). Then, the percentage of apoptotic cells was analysed by flow cytometry. FlowJo software was used for quantitative analysis of the rate of apoptotic cells.

### Statistical analysis

R software (version 3.6.0) and GraphPad Prism (version 9.0) were applied for statistical analysis. The statistical significance of survival analysis was calculated using the log-rank test. The results of qRT-PCR were analysed by the 2−∆∆C method. In addition, statistical deference among subgroups was conducted via Student’s t test. The relationship between clinical variables and RECK expression was analysed by the Kruskal–Wallis H test. Correlation analysis was conducted using nonparametric Spearman’s r test. Differential analysis between tumor immunity subclusters and metastasis was conducted by chi-square test. All experiments were performed at least three times and the data were visualized as the mean ± SD. The differences were considered statistically significant at *P* < 0.05.

## Results

### RECK exhibits a low expression pattern in patients with STAD

**Fig. 1:**
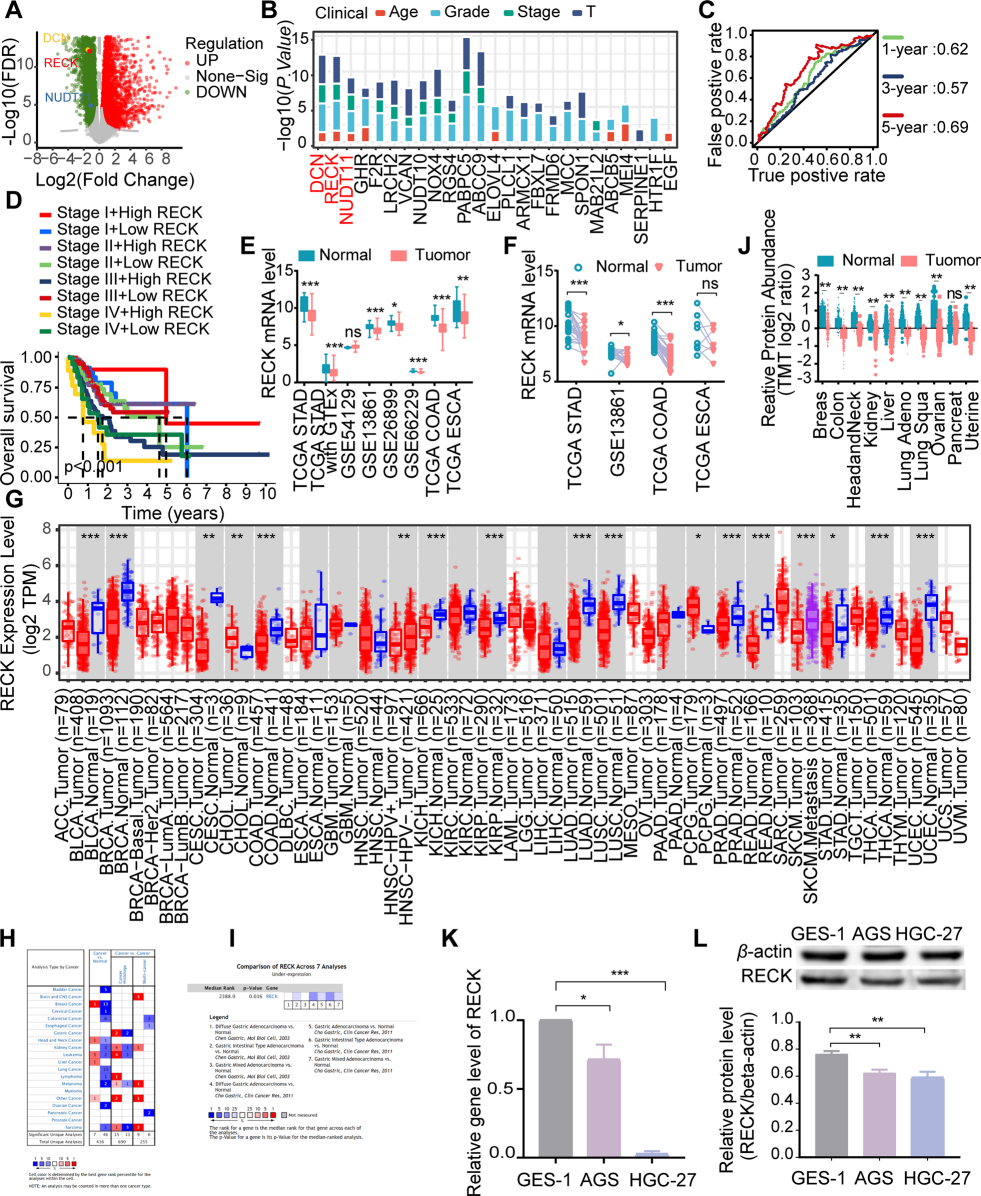
RECK expression is downregulated in gastric and other tumor samples compared to normal samples. **A** Volcano representation demonstrates differentially expressed genes (DEGs) between normal and tumor tissues in the TCGA STAD dataset. An adjusted p-value < 0.05 and |log2(fold change) > 1 were the cut-off criteria. **B** Correlation analysis between tumor-associated genes and clinical characteristics. **C** Time-dependent ROC curve analysis for survival prediction by RECK expression level in patients with STAD. **D** Combination of higher RECK expression and stage I predicted longer OS in the TCGA STAD dataset. **E**–**J** RECK expression was downregulated among tumor samples based on unpaired (**E**) and paired (**F**) difference analyses involving multiple datasets, pan-cancer differential analysis in TIMER2.0 (**G**) and Oncomine (**H**), meta-analysis in Oncomine (**I**), and protein differential analysis in CPTAC (**J**). **K**, **L** Quantitative real-time PCR (qRT-PCR) (**K**) and Western blot analysis (**L**) illustrate the significant downregulation of RECK expression in AGS and HGC-27 cells compared with GES-1 cells (*p < 0.05, **p < 0.01, ***p < 0.001, ns = not significant)

A total of 4208 DEGs were observed in TCGA STAD datasets (Fig. 1A). Subsequently, RECK was screened as a tumor-related candidate gene using multiple bioinformatics strategies with the criterion of *P* < 0.05, including correlation analysis for clinical characteristic relevance, survival analysis, and receiver operating characteristic curve (ROC) analysis. RECK was significantly associated with patient age, tumor grade, stage, and T classification of STAD (Fig. 1B). In addition, the area under curve (AUC) values for RECK were 0.615, 0.571, and 0.688, for predicting 1-, 3-, and 5-year survival rates, respectively (Fig. 1C). GSE13861 and GSE28541 were also used to access the AUC values for RECK (Additional file 2: Fig. S1). The findings suggested that RECK exhibited superior predictive efficacy for long-term outcomes, while its performance in terms of short-term predictive efficacy may be unsatisfactory. Overall survival (OS) analysis indicated that higher RECK expression combined with stage I predicted longer OS in the TCGA STAD dataset (Fig. 1D). Furthermore, unpaired and paired difference analyses illustrated that RECK was downregulated in the tumor samples (Fig. 1E, F). Pan-cancer analysis and meta-analysis of RECK expression from the Oncomine and TIMER2.0 platform yielded consistent results (Fig. 1G–I). Additionally, we observed the same low expression of RECK protein levels in pan-cancer datasets from the CPTAC database (Fig. 1J). The observation that RECK had downregulated expression in STAD was further verified in AGS and HGC-27 cell lines by qRT-PCR and Western blot assays (Fig. 1K, L). The findings of this study indicate that the expression of RECK is significantly reduced in gastric cancer.

### Upregulation of RECK has adverse influences on aggressive tumor behaviors and predicts superior prognosis of patients with GC

**Fig. 2:**
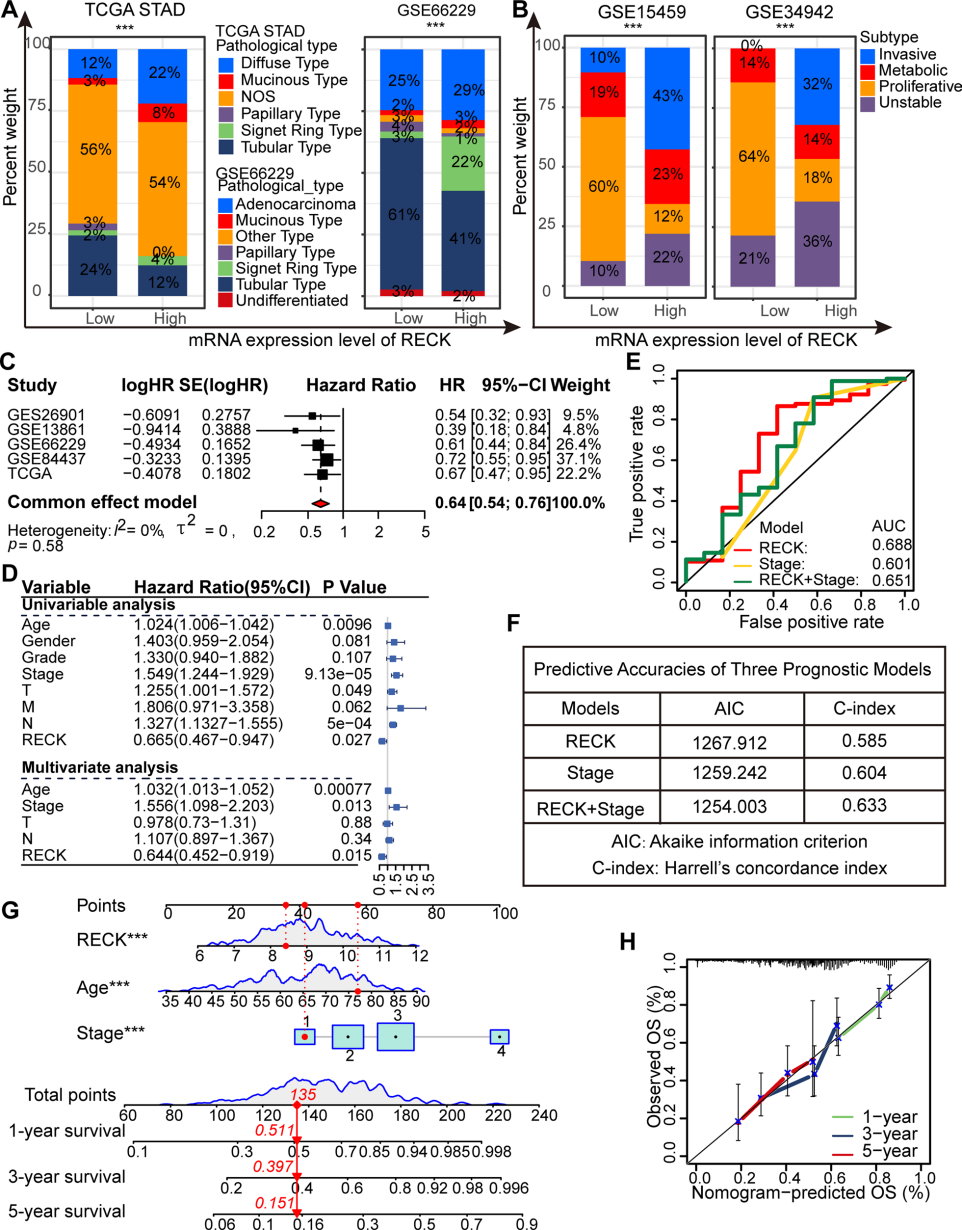
RECK is an independent prognostic factor in patients with gastric cancer. **A**, **B** Comparison of the RECK expression pattern across various datasets with the gastric pathogenic types (**A**) and subtypes (**B**). **C** The meta-analysis confirmed that RECK expression was identified as a protective indicator that might affect the overall survival of patients with gastric cancer. **D** Univariate and multivariate Cox analyses were utilized to identify independent prognostic factors in patients with gastric cancer. **E** ROC analysis for the predictive value of RECK expression model, Stage model, and the combined model. **F** The predictive accuracies of RECK expression model, Stage model, and the combined model were compared by AIC and C-index. **G** A nomogram was performed to quantify the integrated effect of the proven independent prognostic factors for overall survival. **H** Calibration plot of the nomogram for 5-year overall survival (*p < 0.05, **p < 0.01, ***p < 0.001, ns = not significant)

It was evident that an increased incidence of tubular- and proliferation-type GC is more likely to arise when RECK is expressed inadequately (Fig. 2A, B). According to a fixed model of the meta-analysis (significant heterogeneity: p > 0.05, I2 < 50%), higher RECK expression in GC patients distinctly correlated with superior overall survival (Fig. 2C). To further screen independent prognostic factors, hazard ratio (HR), a value from a power of e using the coefficient of a variable in the COX regression model, was utilized. Considering the practical significance of HR values in clinical investigations, the variable is a risk factor when the coefficient is greater than 0 (HR > 1) and a protective factor when the coefficient is less than 0 (HR < 1). According to the univariate and multivariate Cox regression analyses of this study, age (HR = 1.032, 95% CI 1.006–1.042, p = 0.00077), stage (HR = 1.556, 95% CI 1.098–2.203, p = 0.013) and RECK expression (HR = 0.644, 95% CI 0.452–0.919, p = 0.015) were identified as independent prognostic factors for overall survival in gastric cancer patients (Fig. 2D). To provide a more accurate predictive model for the outcomes of GC patients, we developed three models: RECK expression alone, stage alone, and integration of RECK expression with stage. ROC analysis for comparing the predictive ability of the three models illustrated that RECK alone exhibited the highest prognostic ability (AUC: 0.688, 95% CI 0.503–0.872) (Fig. 2E). In addition, the model incorporating RECK expression and stage demonstrated the lowest AIC and the highest C-index (Fig. 2F). A quantitative strategy for more precise overall survival prediction in GC patients was developed using a nomogram that included all proven independent prognostic factors, including age, stage, and RECK expression (Fig. 2G). The quantitative model performed well in the calibration plot for this nomogram’s prediction of 1-year, 3-year and 5-year overall survival (Fig. 2H). These results indicated that RECK could be a promising independent prognostic indicator.

**Fig. 3:**
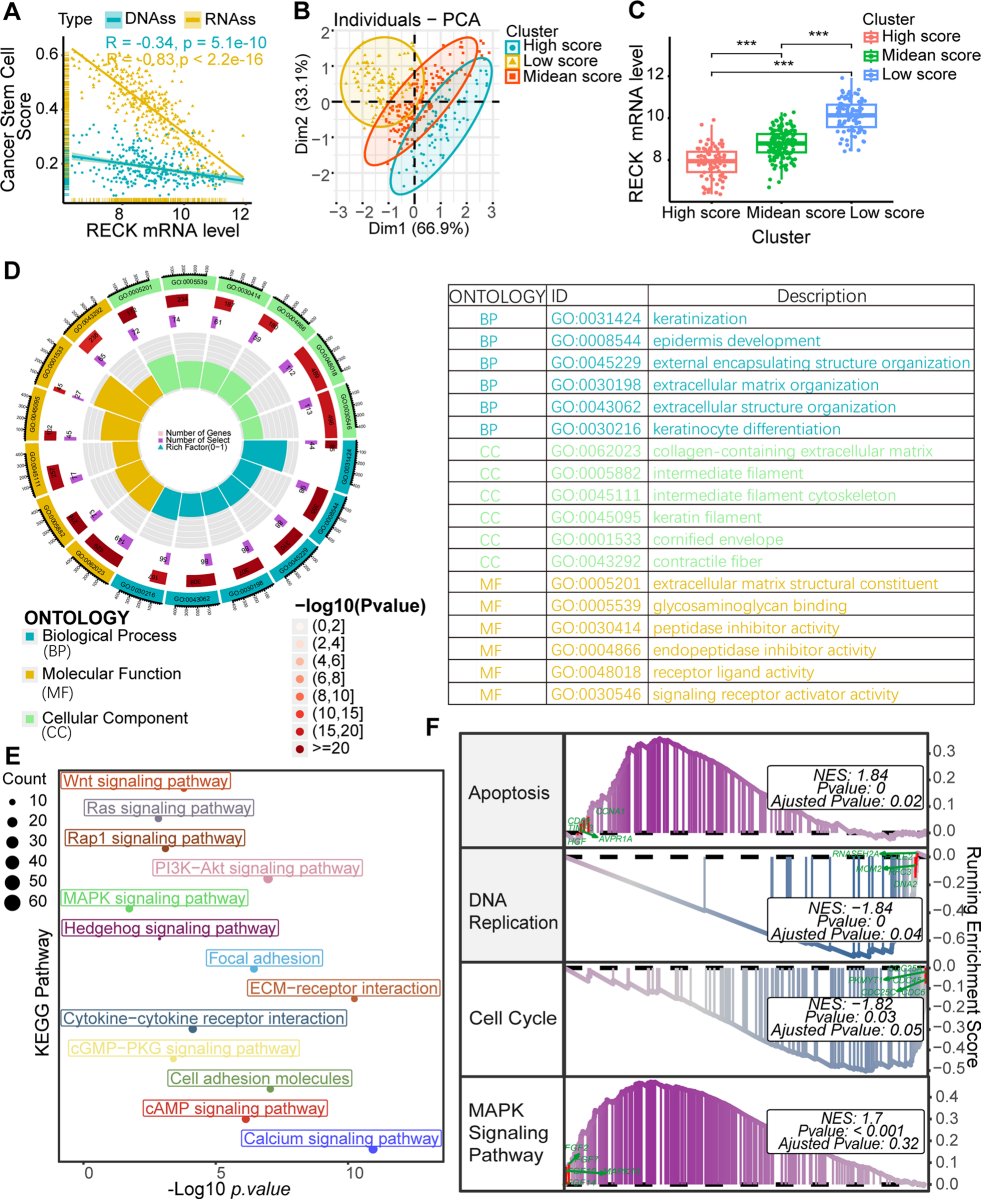
Function enrichment analysis and correlation analysis between RECK expression and cancer stem cells (CSCs) scores. **A** RECK was negatively correlated with CSC scores in TCGA STAD datasets. **B** Principal component analysis (PCA) displayed three distinct subclusters of TCGA STAD patients based on the CSC scores: the high, medium, and low score groups. **C** RECK expression levels decreased concurrently with increased stem cell scores. **D**–**F** Gene Ontology (GO), Kyoto Encyclopedia of Genes and Genomes (KEGG) enrichment analysis, and Gene Set Enrichment Analysis (GSEA) for differentially expressed genes between the high-RECK and low-RECK groups (*p < 0.05, **p < 0.01, ***p < 0.001, ns = not significant)

According to relevant research, the degree of similarity between tumor cells and stem cells can be quantified using two indexes: DNA methylation- and mRNA expression-based stemness index (mDNAsi and mRNAsi) [14], which are known as cancer stem cell scores. The index ranges from 0 to 1, with values near to 1 indicating that the cancer cells have stronger stem cell signature and the lower degree of cell differentiation. CSCs promote tumour progression, metastasis, drug resistance, and enhanced self-renewal via self-protective mechanisms such as DNA damage repair, inhibition of apoptotic pathways, and production of drug-resistant proteins, in contrast to tumour cells lacking stemness. To gain additional insights into the prospective functions of RECK, we investigated the potential association of RECK with mRNAsi in the TCGA STAD dataset. The correlation analysis presented the distinctively negative relevance of RECK with the CSC scores (Fig. 3A). Furthermore, three subgroups were identified based on the CSC scores using hierarchical cluster analysis and principal component analysis (PCA) analysis (Fig. 3B), and the representation displayed that increased RECK expression levels were accompanied by decreased stem cell scores (Fig. 3C). These findings indicated that patients with high expression of RECK may be associated with low CSC scores, implying that these patients may exhibit improved treatment outcomes and better prognosis.

Additionally, the TCGA STAD cohort was divided into two groups based on the median of RECK expression. The differential analysis identified a total of 2187 DEGs between the high and low RECK groups under the same criteria described above; subsequently, enrichment analyses were conducted using these DEGs. The results of GO, KEGG, and GSEA suggested that RECK might be involved in several crucial pathways underlying cancer progression, including MAPK signaling, Wnt signaling, apoptosis, DNA replication, and the cell cycle, as well as other functional pathways (Fig. 3D–F). MAPK signaling, Wnt signaling, apoptosis, DNA replication, and the cell cycle are frequently observed to be aberrantly enriched in the genesis of cancer. The findings of this study indicate a strong association between RECK expression and the enriched cancer-related pathways, which may potentially contribute to the anticancer effects of RECK.

### High RECK expression shows a remarkably positive relationship with a high immunity status

**Fig. 4:**
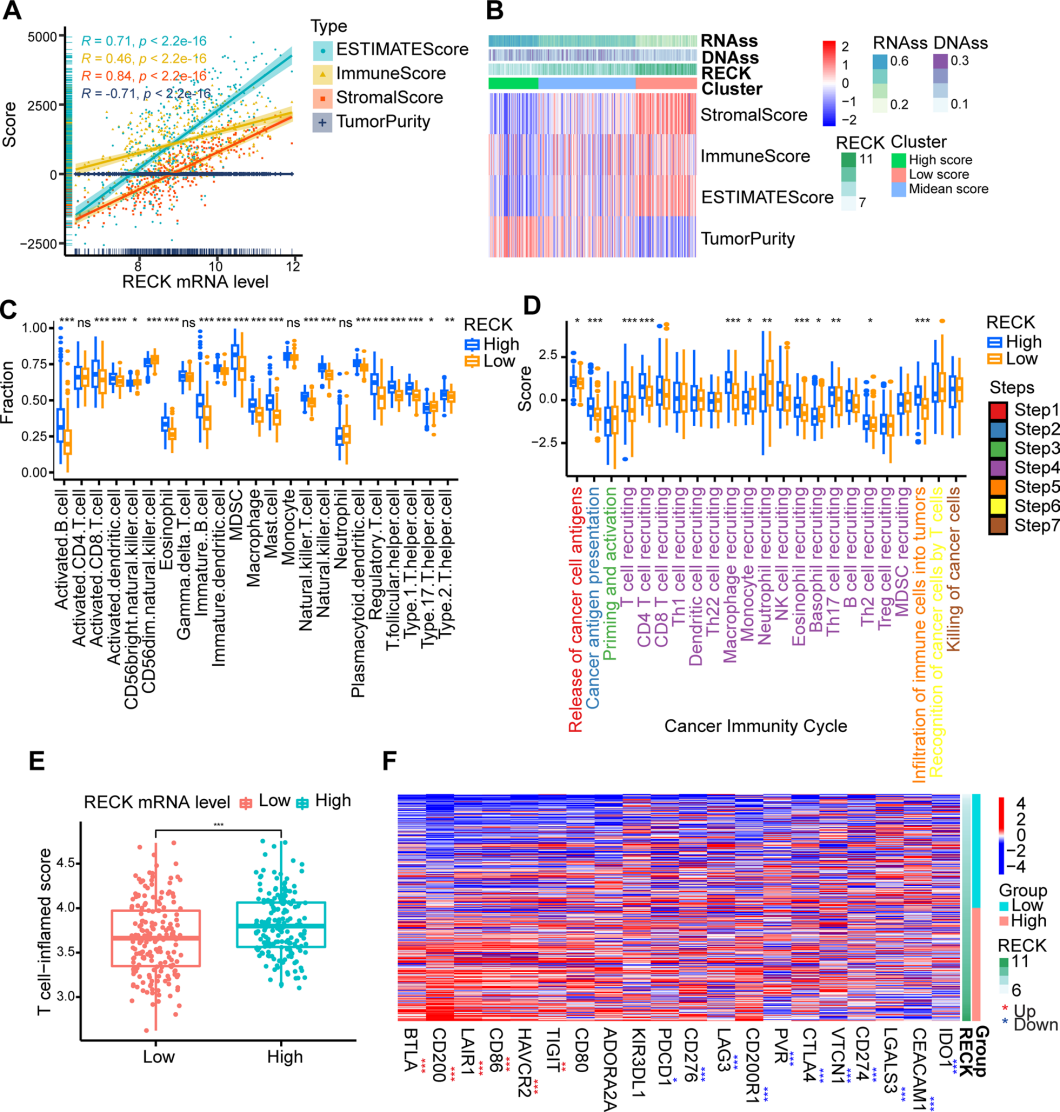
Correlation analysis of RECK with the immunity status of the tumor microenvironment in gastric cancer tissues. **A** Relevance of RECK expression in the TCGA STAD dataset to the tumor microenvironment. **B** The heatmap indicates the relationship between the CSCs’ score and the tumor microenvironment in the TCGA STAD dataset. **C**, **D** Differential analysis for the infiltration level of the TIICs calculated by single sample Gene Set Enrichment Analysis (ssGSEA) (**C**) and the levels of cancer immunity cycle (**D**) between the high- and low-RECK groups in the TCGA STAD dataset. **E** High RECK expression was accompanied by a high T cell-inflamed score in the TCGA STAD dataset. **F** Heatmap for correlational analysis of RECK with the level of inhibitory immune checkpoints in tumor samples derived from TCGA STAD dataset (*p < 0.05, **p < 0.01, ***p < 0.001, ns = not significant)

Based on the findings above, we investigated the potential linkages between RECK expression, tumor immunity, and metastasis since CSCs were closely related to tumor immunity status and metastasis. To explore the correlation between RECK expression and the tumor microenvironment, the “ESTIMATE” R package was utilized to compute an estimate score (the combination of stromal score and immune score) and the tumor purity. The stromal score captures the presence of stroma in tumor tissue, whereas the immune score represents the infiltration of immune cells in tumor tissues. Additionally, the tumor purity reflects the proportion of tumor cells inside a tumor sample. The correlation analysis illustrated that RECK was positively correlated with the stromal score, immune score, and estimate score; however, tumor purity exhibited a significantly negative relationship with RECK expression (Fig. 4A). Moreover, the heatmap depicting the dominant association of CSCs, the tumor microenvironment, and RECK expression was shown in Fig. 4B. Subsequently, the relationship between RECK expression and the levels of immune cell infiltration was investigated. As determined by the correlation analysis between the levels of infiltrating immune cells calculated using 7 algorithms and RECK expression, infiltrating levels of CD8^+^ T cells, CD4^+^ T cells, monocytes, and macrophages were positively correlated with RECK expression (Table 1). The levels of infiltrating immune cells from single sample Gene Set Enrichment Analysis (ssGSEA) confirmed the concordant results as described previously (Fig. 4C). CD8^+^ T cells possess the ability to play a crucial function in the eradication of malignant cells and CD4^+^ T cells primarily function as immune memory and immune protection during tumor metastasis. The observed positive connection between the expression of RECK and the presence of CD8+ or CD4+ T cells implies that the GC patients exhibiting high levels of RECK may also exhibit a higher abundance of T cells in tumor environment, hence potentially indicating a more favorable prognosis. The activities of the cancer immunity cycle also confirmed that T-cell recruitment and CD4^+^ T-cell recruitment from step 4 were upregulated in the high RECK expression group (Fig. 4D), as well as infiltration of immune cells into tumors. Elevated RECK expression implies enhanced immune response, which may render the individual more receptive to anti-tumor therapeutic interventions. Owing to the potential association between RECK and T cells, the T-cell-inflamed score was calculated (Additional file 1: Table S3), and the relationship of the T-cell-inflamed score with RECK expression exhibited consistently positive relevance (Fig. 4E). Subsequently, differential analysis revealed that PD-1 (PDCD1) and PD-L1 (CD274) were downregulated in the high RECK group, whereas TIGIT and BTLA were upregulated (Fig. 4F, Additional file 1: Table S4). The expression level of various immune checkpoints influences the selection of subsequent immunotherapy strategies in cancer treatment. Based on the results of this study, immunotherapy against TIGIT and BTLA was preferable to immunotherapy against PD-1and PD-L1 for GC patients with elevated RECK expression. These results indicated that high RECK is distinctly relevant to high immunity status in the tumor microenvironment, especially CD4^+^ T cells and B cells. Furthermore, the assessment of RECK expression levels holds promise as a possible indicator for guiding immunotherapy strategies in patients with gastric cancer.

**Table 1:** Correlational analysis of RECK expression with 6 types of tumor-infiltrating immune cells calculated by 7 different algorithms

Immune cells	CIBERSORT	CIBERSORT-ABS	EPIC	MCPCOUNTER	QUANTISEQ	TIMER	XCELL
CD8^+^T cell	ns	0.33	ns	0.19	0.22	0.37	0.2
CD4^+^T cell	− 0.15	0.54	0.29	0.25	0.25	0.48	0.34
NK cell	− 0.15	0.2	ns	0.3	ns	ns	ns
Macrophage	M0:0.19 M2: 0.23	M1:0.23 M2: 0.57	0.37	0.48	M2:0.6	0.7	M0:0.18 M1:0.21 M2: 0.2
Monocyte	0.25	0.43	ns	0.48	0.18	ns	0.35
Neutrophil	− 0.1	ns	ns	0.2	ns	0.48	ns

The value represents the correlation coefficient determined by Spearman’s r test with *P* < 0.05

*ns* not significant

**Fig. 5:**
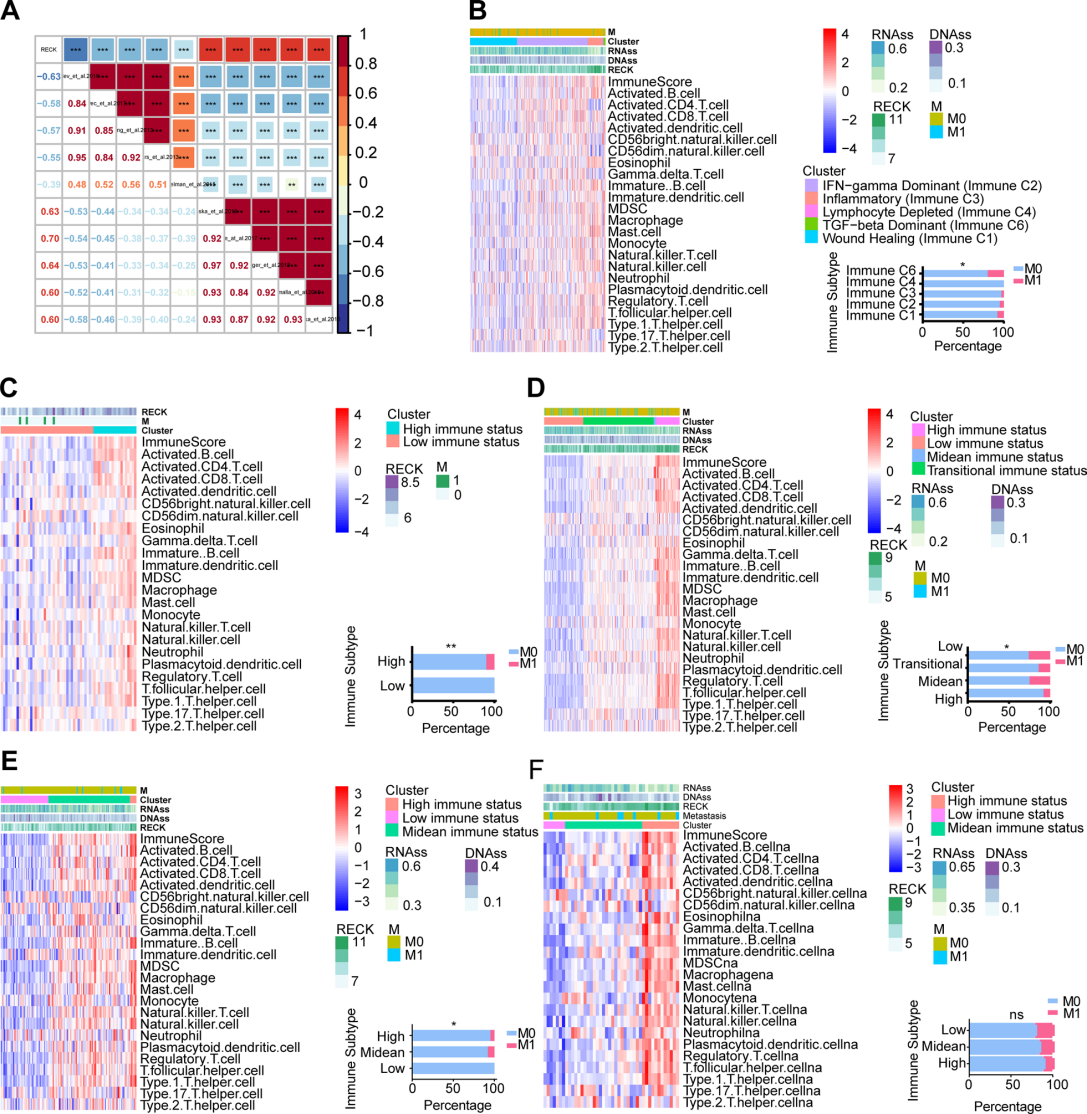
RECK might mediate the cross-talk between tumor immunity status and tumor metastatic capacity in patients with STAD. **A** Correlation analysis of RECK with metastasis-related gene sets. **B**–**F** Higher immunity status in tumor samples accompanied by a lower rate of tumor metastasis was demonstrated in the TCGA STAD (**B**), GSE13861 (**C**), TCGA COAD (**D**), and TCGA ESCA (**E**) datasets, except for TCGA READ (**F**) (*p < 0.05, **p < 0.01, ***p < 0.001, ns = not significant)

Gene sets from the EMTome platform were used to score each gene set by GSVA. The correlation analysis between RECK expression and gene set score indicated that RECK is significantly correlated with EMT in GC patients (Fig. 5A). Subsequently, we calculated the infiltrating levels of tumor immune cells using ssGSEA across several datasets, including the GSE13861, TCGA COAD, TCGA ESCA, and TCGA READ datasets. Based on the infiltrating levels of tumor immune cells, patients were divided into several immune subtypes, and differential analysis across these immune subtypes revealed that higher immune status was accompanied by a lower metastasis rate, expect for GSE13861 and TCGA READ (Fig. 5B–E). Hence, these results suggested that high RECK expression might be relate to a high-immunity-status microenvironment and a reduced metastasis rate in GC patients and might be a link between a high-immunity-status microenvironment and a lower metastasis rate.

### RECK exerts a tumor suppressor on inhibiting cell proliferation by regressing the G1-S cell cycle and inducing cell apoptosis

**Fig. 6:**
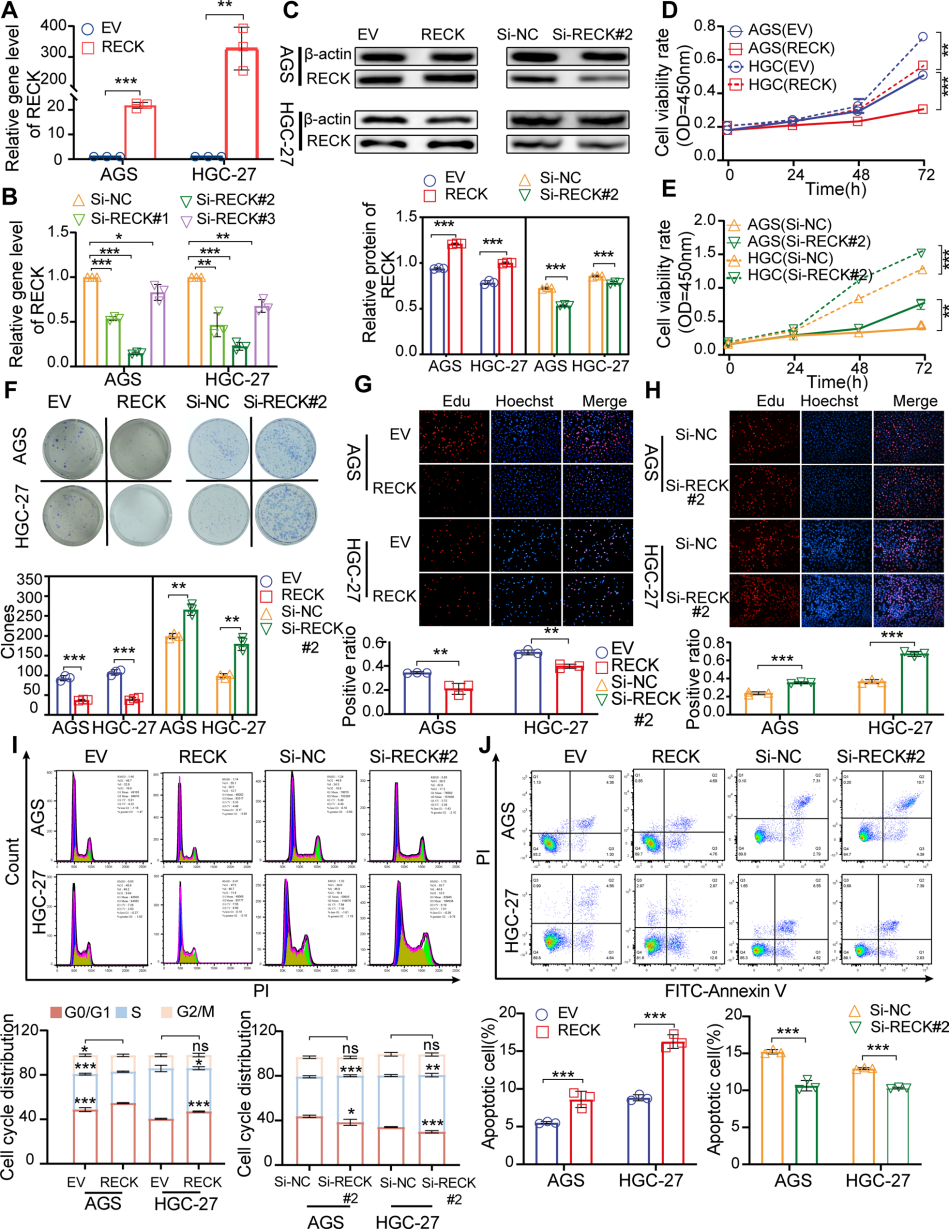
RECK influences GC cell proliferation via regulating the cell cycle and apoptosis in GC cells. **A**–**C** RECK knockdown and overexpression were detected by qRT-PCR (**A**, **B**) and western blot assays in AGS and HGC-27 cells (**C**). **D**–**H** CCK-8 (**D**, **E**), colony formation assay (**F**), and EdU assay (**G**, **H**) were applied for cell proliferation analysis in AGS and HGC-27 cells after RECK overexpression or knockdown. **I**, **J** Cell cycle (**I**) and cell apoptosis (**J**) were detected by flow cytometry in AGS and HGC-27 cells following overexpression or knockdown of RECK (*p < 0.05, **p < 0.01, ***p < 0.001, ns = not significant)

**Fig. 7:**
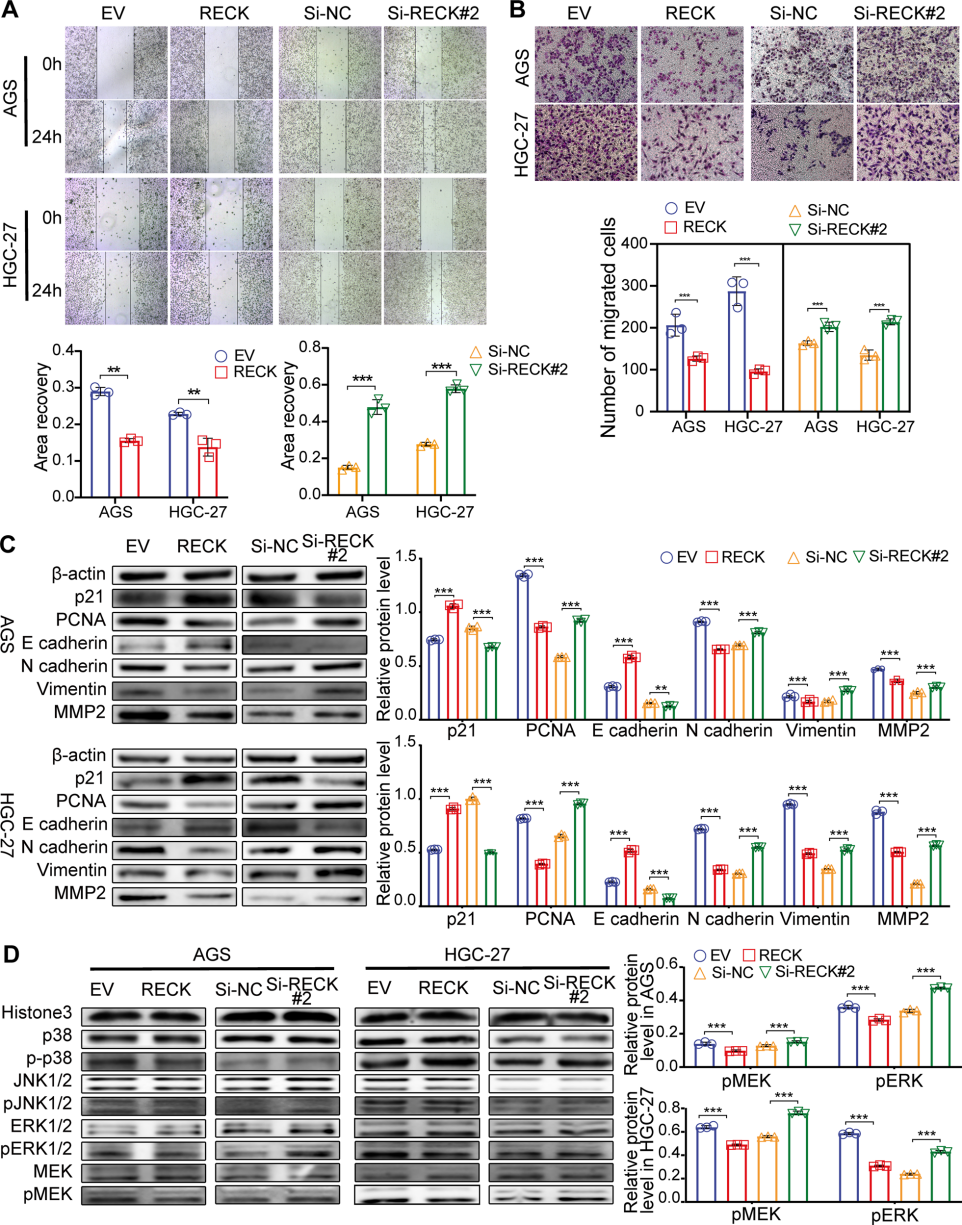
RECK modulates the migration and invasion of GC cells as well as ERK/MAPK signaling. **A** The migration capacity of GC cells with RECK overexpression or knockdown in AGS and HGC-27 cells was evaluated through the wound-healing assay. **B** The invasion capacity of GC cells with RECK overexpression or knockdown in AGS and HGC-27 cells was measured through Matrigel invasion assay. **C**, **D** The effects of RECK overexpression or knockdown on cell proliferation markers (**C**), EMT markers (**C**), and MAPK signaling markers (**D**) in AGS and HGC-27 cells were detected by western blot assay (*p < 0.05, **p < 0.01, ***p < 0.001, ns = not significant)

To elucidate the effect of RECK on the malignant phenotypes of GC cells, RECK was overexpressed by plasmid-mediated transduction and knocked down by RNA interference in AGS and HGC-27 cells, and the efficiencies were verified by qRT-PCR and western blot assays (Fig. 6A–C). CCK-8, colony formation and EdU assays demonstrated that RECK overexpression remarkably hampered the growth of AGS and HCG-27 cells, while RECK knockdown stimulated cell proliferation (Fig. 6D–H, Additional file 2: Fig. S2). The effect of RECK on cell cycle distribution was explored to determine the molecular mechanism by which RECK inhibited GC cell proliferation. Upregulation of RECK induced effective cell cycle arrest of the G1-to-S cell phase in AGS and HGC-27 cells. In contrast to the effects of RECK overexpression, RECK knockdown displayed the opposite implication on cell cycle progression (Fig. 6I). To observe the molecular variation following RECK dysfunction, proliferation-related proteins were examined by western blot assay. The protein level of p21, cyclin dependent kinase inhibitor 1A, was elevated in response to RECK overexpression and decreased in response to RECK knockdown (Fig. 7C). The protein level of PCNA, proliferating cell nuclear antigen, was reduced by RECK overexpression and increased by RECK knockdown (Fig. 7C). In addition, the cell apoptosis assay for AGS and HGC-27 cells revealed more apoptotic cells in RECK-overexpressed cells and fewer apoptotic cells in RECK-knockdown cells than in control cells (Fig. 6J).

### RECK exerts a tumor suppressor on cell migration and invasion by inhibiting the EMT process in GC cells

We performed wound-healing migration and transwell invasion assays to evaluate the implication of RECK on the metastatic potential of GC cells. Overexpression of RECK significantly inhibited the migration and invasion of AGS and HGC-27 cells, whereas knockdown of RECK distinctly enabled the migration and invasion of AGS and HGC-27 cells (Fig. 7A, B). To expound the potential mechanism of migration and invasion mediated by RECK in GC cells, the protein levels of EMT markers were examined. The results in AGS and HGC-27 cells indicated that RECK overexpression significantly increased the protein levels of epithelial markers (E-cadherin, CDH1) while decreasing the levels of mesenchymal markers (N-cadherin (CDH2) and Vimentin) and the prometastatic metalloproteases MMP2 (Fig. 7C). The upregulation of N-cadherin, Vimentin and MMP2 facilitates the process of EMT and contributes to the metastasis of tumor cells. Conversely, the upregulation of E-cadherin exhibits an opposing impact. Therefore, the overexpression of RECK had the capacity to modulate the expression of these proteins, including E-cadherin, N-cadherin, Vimentin and MMP2, thereby exerting inhibitory effects on the migration and invasion of tumor cells. However, RECK knockdown displayed the opposite effects on EMT markers compared to RECK overexpression (Fig. 7C). These findings suggested that RECK hampered the expression of EMT genes to regress the migration and invasion of GC cells.

### High RECK decreases ERK phosphorylation in GC cells

Additionally, MAPK signaling, resulting from KEGG enrichment, was examined by western blot assay in AGS and HGC-27 cells with up- or down-regulated RECK. The findings confirmed that RECK expression regulated the phosphorylation of ERK but not JNK or p38 in MAPK signaling (Fig. 7D).

### ERK phosphorylation mediates RECK-induced inhibition of cell proliferation and the EMT process

**Fig. 8:**
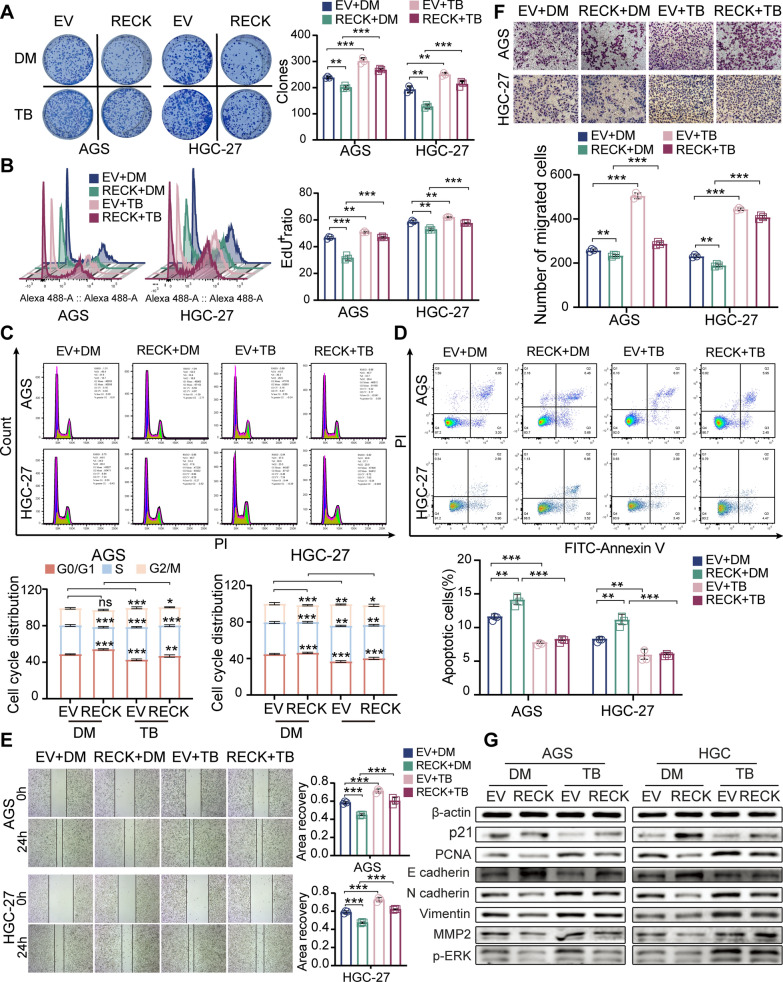
TBHQ, an ERK activator, rescued the inhibitory phenotype of GC cells by RECK overexpression. **A**, **B** Colony formation assay (**A**) and EdU assay (**B**) for GC cell proliferation evaluation in RECK-overexpressed AGS and HGC-27 cells treated with TBHQ. **C**, **D** Cell cycle (**C**) and cell apoptosis (**D**) were detected by flow cytometry in RECK-overexpressed AGS and HGC-27 cells treated with TBHQ. **E**, **F** The migration and invasion abilities of GC cells were measured using wound-healing and Matrigel invasion assays in RECK-overexpressed AGS and HGC-27 cells treated with TBHQ. **G** Western blot representations of cell proliferation markers, EMT markers, and MEK/ERK signaling markers levels in RECK-overexpressed AGS and HGC-27 cells treated with TBHQ (*p < 0.05, **p < 0.01, ***p < 0.001, ns = not significant. DM: DMSO; TB: TBHQ)

**Fig. 9:**
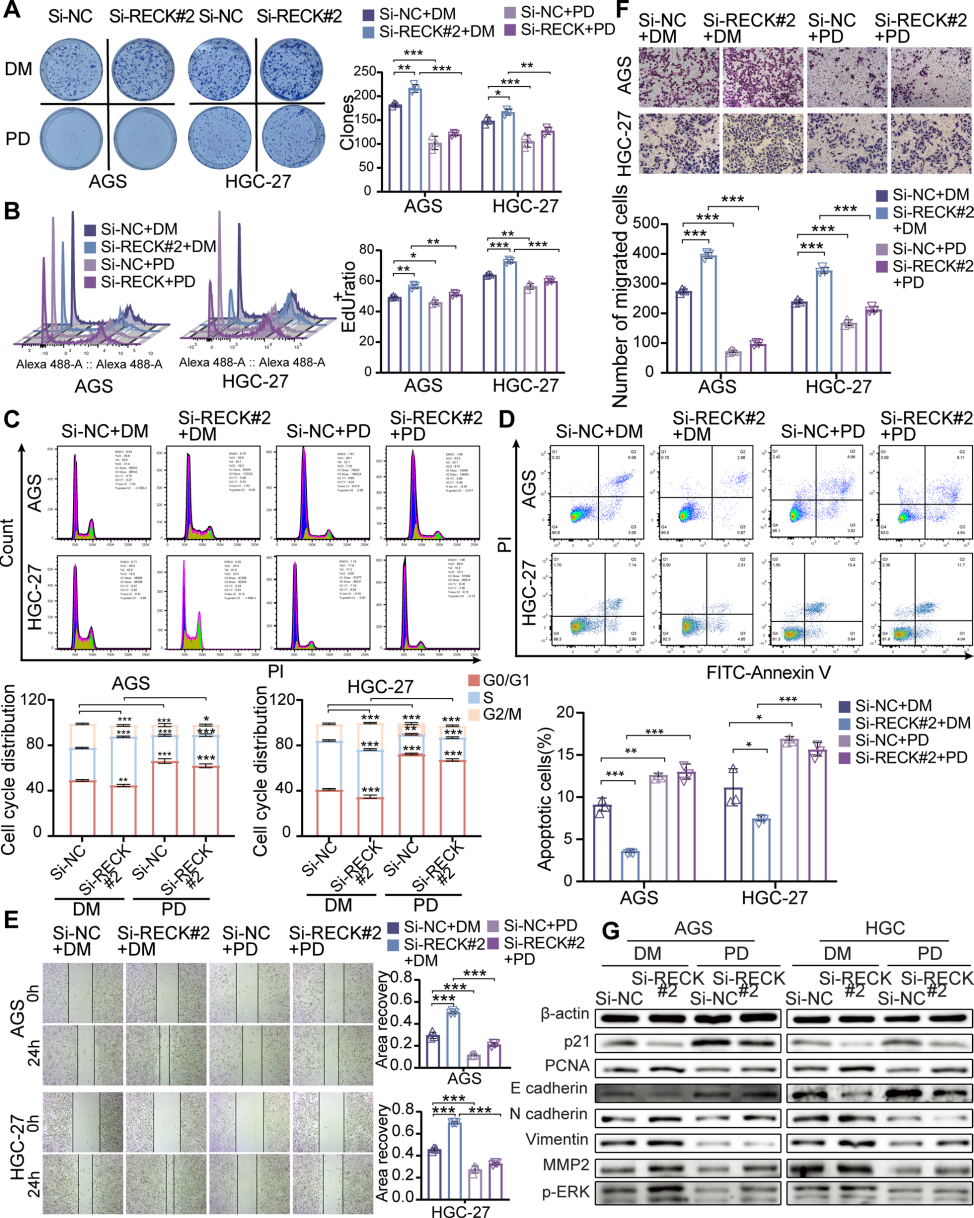
PD98059, an ERK inhibitor, inhibited the stimulatory phenotype of GC cells by RECK knockdown. **A**, **B** Colony formation assay (**A**) and EdU assay (**B**) for GC cell proliferation evaluation in RECK-knockdown AGS and HGC-27 cells treated with PD98059. **C**, **D** Cell cycle (**C**) and cell apoptosis (**D**) were detected by flow cytometry in RECK-knockdown AGS and HGC-27 cells treated with PD98059. **E**, **F** The migration and invasion abilities of GC cells were measured using wound-healing and Matrigel invasion assays in RECK-knockdown AGS and HGC-27 cells treated with PD98059. **G** Western blot representations of cell proliferation markers, EMT markers, and MEK/ERK signaling markers levels in RECK-overexpressed AGS and HGC-27 cells treated with PD98059 (*p < 0.05, **p < 0.01, ***p < 0.001, ns = not significant. DM: DMSO; PD: PD98059)

To further investigate whether the RECK-induced obstacle of growth and metastasis is caused by modulation of ERK/MAPK signaling, GC cells were treated with an ERK inhibitor, PD98059, and an ERK activator, TBHQ. TBHQ treatment remarkably abrogated the RECK-mediated reduction in cell proliferation in RECK-upregulated AGS and HGC-27 cells via colony formation, EdU, and cell cycle flow cytometry assays (Fig. 8A–C). Activation of ERK phosphorylation by TBHQ significantly reversed RECK overexpression-induced upregulation of p21 and downregulation of PCNA in AGS and HGC-27 cells (Fig. 8G, Additional file 2: Fig. S3A, B). In addition, TBHQ treatment distinctly decreased RECK-overexpression-mediated upregulation of the cell apoptosis percentage in AGS and HGC-27 cells (Fig. 8D). The inhibitory influence of RECK overexpression on the migratory, invasive capacities and EMT process in AGS and HGC-27 cells was also hampered by TBHQ treatment (Fig. 8E–G, Additional file 2: Fig. S3A, B). Similar to the findings mentioned above, PD98059 treatment obviously reversed the pro-tumor influences of RECK knockdown on cell proliferation and the EMT process (Fig. 9, Additional file 2: Fig. S3C, D). Hence, these findings implied that the antiproliferative and anti-metastatic impacts of RECK in gastric cancer were ERK-dependent.

### RECK promotes CALD1 expression and inactivation of CALD1 phosphorylation by hampering ERK1/2 phosphorylation

**Fig. 10:**
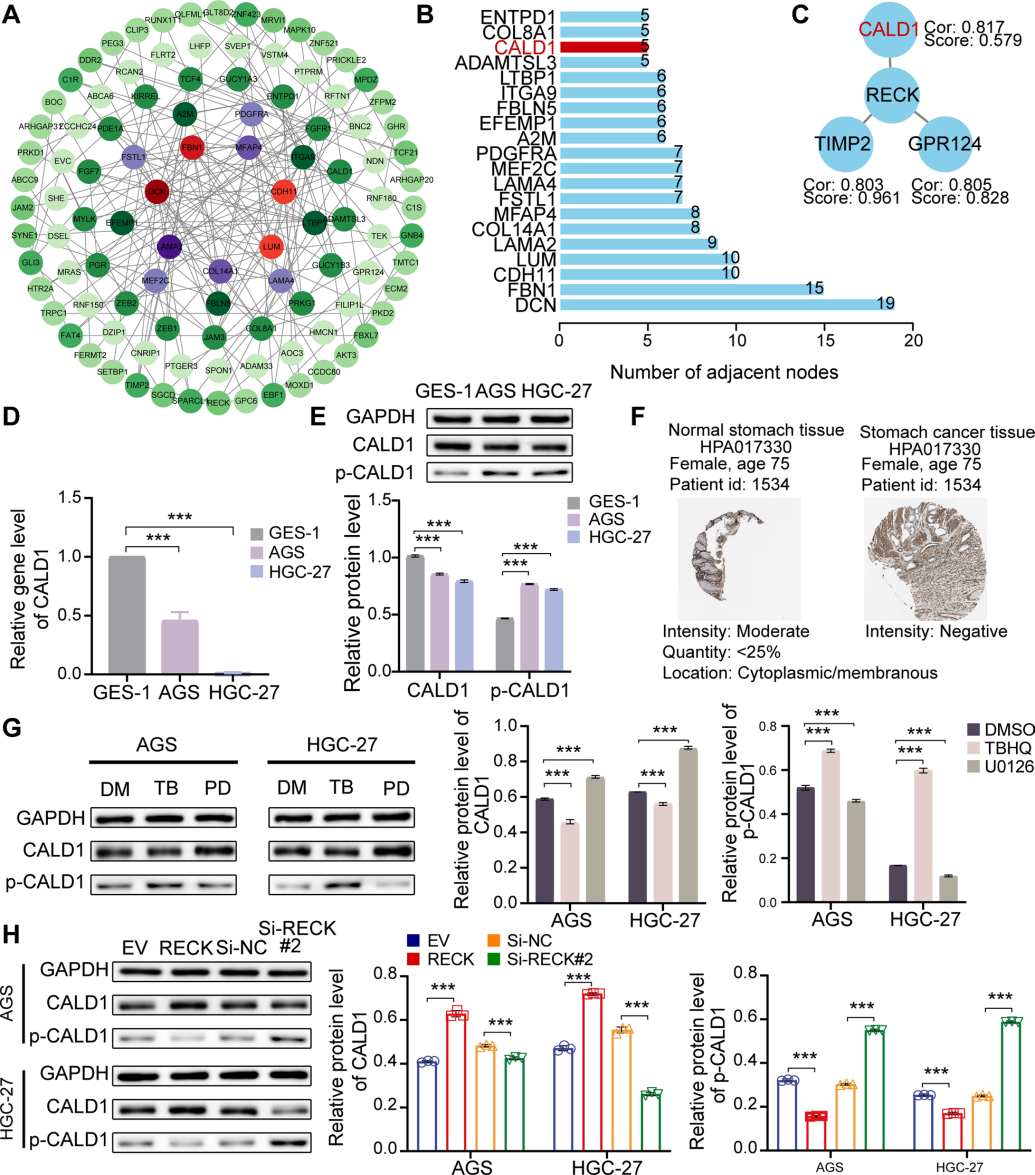
RECK modulates the expression level of CALD1 via influencing the phosphorylation level of ERK. **A** The PPI network based on genes associated with RECK was constructed by the STRING platform and Cytoscape 3.9 software. **B** The bar plot depicts the number of adjacent nodes per gene according to the STRING platform. **C** Three genes from the PPI network were directly related to RECK. Cor: the coefficient for correlation analysis. Score: combined score derived from STRING platform. **D**–**F** The downregulation of CALD1 in GC was verified by qRT-PCR, Western blot analysis, and immunohistochemical assay from the Human Protein Atlas (HPA). **G** The phosphorylation level of CALD1 was performed by Western blot analysis in AGS and HGC-27 cells with the treatment of TBHQ or PD98059. **H** The effect of alterations in RECK gene expression on the phosphorylation level of CALD1 in AGS and HGC-27 cells (*p < 0.05, **p < 0.01, ***p < 0.001, ns = not significant)

Correlation analysis was utilized to identify genes associated with RECK, and 172 genes were selected with the criteria of correlation coefficient ≥ 0.8 and p < 0.05. A protein-protein interaction (PPI) network was constructed for 172 genes via the STRING platform and visualized by Cytoscape software (Fig. 10A). The top 30 genes with the highest degrees of connectivity were shown in Fig. 10B. Moreover, the representation illustrated three genes directly related to RECK, including CALD1, TIMP2 and GPR124 (Fig. 10C). Taking into account connectivity degrees, correlation coefficients and research reports [15], CALD1 was selected cautiously for further research. Subsequently, the significant downregulation of CALD1 was verified by qRT-PCR, western blot assays, and the HPA platform (Fig. 10D–F). In concordance with previous literature, TBHQ treatment resulted in a distinct decrease in CALD1 expression and an increase in CALD1 phosphorylation in AGS and HGC-27 cells (Fig. 10G). PD98059 treatment had opposing influences compared to TBHQ’s effects (Fig. 10G). These results indicated that ERK phosphorylation levels remarkably modulated CALD1 phosphorylation levels. To investigate RECK’s regulation on CALD1, we examined the protein levels of CALD1 and CALD1 phosphorylation. As expected, RECK overexpression facilitated the CALD1 expression level and inactivated the CALD1 phosphorylation level in AGS and HGC-27 cells (Fig. 10H). The treatment of GC cells with RECK suppression had opposite effects on CALD compared to RECK overexpression (Fig. 10H). The alterations found in CALD1 and CALD1 phosphorylation, which were triggered by RECK, may potentially contribute to the anticancer actions of RECK in gastric cancer cells. These results indicated that RECK might facilitate the CALD1 expression level and inactivation of CALD1 by inhibiting ERK1/2 phosphorylation. Concurrently, the mechanism, involving the interplay between ERK1/2 and CALD1, appeared to contribute to the anticancer effects of RECK in gastric cancer.

### CALD1 mediates RECK-induced inhibition of cell proliferation and the EMT process in GC cells

For a deeper understanding of the f CALD1 expression on the anticancer properties of RECK in GC, co-transfection experiments including the transfection of RECK and CALD1, were performed by recombinant plasmid and RNA interference. The verification of CALD1 knockdown efficiency was conducted by the utilization of qRT-PCR and western blot tests (Additional file 2: Fig. S4A, B). The downregulation of CALD1 resulted in the significant elimination of the RECK overexpression-induced decrease in cellular proliferation of AGS and HGC-27 cells, as demonstrated by colony formation and cell cycle flow cytometry assays (Additional file 2: Fig. S4C, D). Similarly, the CALD1 downregulation triggered a considerable reversal of RECK overexpression-induced upregulation of p21 and downregulation of PCNA in AGS and HGC-27 cells (Additional file 2: Fig. S4H). Furthermore, the observed increase in the proportion of apoptotic cells in RECK over-expressing AGS and HGC-27 cells was arrested by CALD1 knockdown (Additional file 2: Fig. S4E). The inhibitory influence of RECK overexpression on the migratory, invasive capacities, and EMT process in AGS and HGC-27 cells was also hindered by CALD1 downregulation (Additional file 2: Fig. S4F, G). CALD1 depletion halted the RECK overexpression-induced downregulation of N cadherin, Vimentin, and MMP2 in AGS and HGC-27 cells, as well as the upregulation of E cadherin induced by RECK overexpression (Additional file 2: Fig. S4H). Consistent with the aforementioned findings, CALD1 overexpression clearly counteracted the pro-tumor influences of RECK knockdown in terms of cell proliferation and the EMT process (Additional file 2: Fig. S5). Consequently, these results suggested that the antiproliferative and anti-metastatic impacts of RECK in gastric cancer were CALD1-dependent.

## Discussion

GC is an aggressive malignant tumor for the reason of its heterogeneity and complexity and is frequently diagnosed at an advanced stage, which contributes to poor prognosis in patients with STAD [16]. However, the efficacy of molecular-targeted therapy remains limited and variable [17, 18]. Therefore, the identification of specific and efficient molecular targets for STAD treatment is extremely crucial.

In the current study, the decreased expression of RECK was verified in TCGA and GEO datasets as well as GC cells. In line with our results, RECK expression was significantly downregulated in several kinds of tumors, such as gallbladder, oral squamous, and ovarian and hepatocellular carcinoma [6, 19–21]. Increased REK expression was identified as an independent and significant factor in predicting a superior prognosis in breast, pancreatic, and hepatocellular carcinomas by kinds of reports [5, 7, 8, 22]. In our analysis, RECK expression was remarkably related to the incidence of tubular-type, and proliferation-type GC, as well as clinical characteristics including age, grade, T, and stage. Significantly, RECK upregulation was accompanied by prolonged overall survival in the GC cohort. To further investigate RECK’s impact on the outcomes of GC patients, we compared the predictive efficacy of the three models and discovered that the combination model provided accurate predictive efficacy. However, the predictive value of ERCK alone was higher than that of stage alone. These results suggested that RECK may be involved in the pathogenesis and development of GC and may serve as a biomarker for predicting the outcome of GC patients.

Cancer stem cells are responsible for tumor resistance, recurrence, and metastasis [23]. It has been reported that RECK inhibited stemness gene expression and stem-like properties by repressing Notch 1 activation in GC [24]. Here, our analysis showed a negative association between RECK expression and mRNAsi for cancer stem cell scores, which implied that there was a positive correlation between high expression levels of RECK and a decreased impact of medicine resistance, diminished cancer recurrence, and a more favorable prognosis. A research manifested that upregulation of RECK is related to a high ESTIMATEscore, recruitment of more tumor-infiltrating lymphocytes, lower tumor purity and high PD-L1 expression, and might function as an indicator for ICI treatment in hepatocellular carcinoma [8]. Moreover, RECK overexpression contributed to a trend of increasing lymphoid-like inflammatory infiltrating cells in cervical carcinogenesis [4]. Here, we demonstrated that increasing RECK expression is distinctly associated with the ESTIMATES score, especially CD4^+^ and CD8^+^ T infiltrating cells in the tumor microenvironment of GC. The findings from the immune-related analyses suggested that the expression of RECK has the potential to serve as a predictive marker for immunological status and as a guideline for ICI treatment. In addition, RECK might act as a link between tumor immunity and metastasis in GC in our analysis. These findings evidenced that high RECK expression was accompanied by a high immunity status and a reduced incidence of metastasis in GC.

Subsequently, to investigate the prospect that RECK could influence the carcinogenesis and development of GC, we modulated the expression of RECK in GC cells. The results revealed that RECK significantly repressed cell proliferation, migration, and invasion in RECK-overexpressed and -knockdown GC cells. A large body of evidence confirmed that RECK modulated tumor cell proliferation, migration, and invasion via kins of signaling including p53 signaling, Notch 1 signaling, and Wnt/β-catenin pathways in cervical cancer, non-small cell lung cancer, and human mesenchymal stem cells [24–26]. Here, based on the MAPK signaling identified by enrichment analysis and dysfunction after RECK overexpression or knockdown verified by western blot assay, we designed rescue experiments using an ERK activator and inhibitor. The results manifested that the ERK activator or inhibitor distinctly reversed the antitumour effects of RECK overexpression or the protumour effects of RECK knockdown in GC cells, respectively. According to the findings above, RECK inhibited tumor proliferation, migration, and invasion by inactivating the MAPK/ERK signaling pathway.

Subsequently, we screened three meaningful candidate genes associated with RECK by bioinformatics analysis, including caldesmon 1 (CALD1), TIMP metallopeptidase inhibitor 2 (TIMP2), and adhesion G protein-coupled receptor A2 (ADGRA2 or GPR124). A report illustrated that AHSA1 enhanced EMT and HCC cell proliferation by ERK/ CALD1 axis [15]. In this study, the dysfunctional expression and phosphorylation of CALD1 resulted from alterations in RECK expression or ERK phosphorylation level. This suggested that RECK might exert antitumour effects by CALD1 expression and phosphorylation levels mediated by ERK phosphorylation levels. For further investigating the potential mechanism between RECK and CALD1, co-transfection was conducted in GC cells. These results demonstrated that the CALD1 deletion or upregulation significantly reversed the antitumor effects of RECK overexpression or the protumor effects of RECK knockdown in GC cells, respectively. According to the findings, it was shown that RECK exerted regulatory control on cellular processes such as proliferation, invasion, and migration through the function of CALD1.

This work contributes to the understanding of potential molecular mechanisms involved in the occurrence and development of GC but still has some limitations. First, the inhibitory phenotypes by RECK were lack of verification in vitro. Second, the exact molecular mechanisms by which RECK promoted cell apoptosis and induced the arrest of the G1-S cell cycle remain unknown. Third, the correlation of RECK with tumor immunity status requires additional experimental verification. Fourth, more studies are essential to determine the interaction between RECK and CALD1. Finally, the prognostic role of RECK requires further verification in a large external dataset.

## Conclusion

In summary, our study revealed that RECK was a favorable prognostic factor and acted as a tumor suppressor of tumor cell proliferation, migration and invasion mediated by inactivation of MAPK/ERK signaling. Moreover, the cross-talk between tumor immunity and metastasis might be mediated by RECK. These findings indicated that RECK could be a promising prognostic biomarker and a potential therapeutic target for GC treatment.

### Supplementary Information

**Additional file 1: Table S1.** The sequences of siRNA targeting RECK. **Table S2.** Primer sequences used in RT-qPCR experiments. **Table S3.** The pan-cancer T cell inflamed score of TCGA STAD patients. **Table S4.** Comparisons of the inhibitory immune checkpoints between RECK groups.

**Additional file 2: Figure S1.** Time-dependent ROC curve analysis for survival prediction by RECK expression level in patients with STAD from GSE13861 and GSE28541 datasets. **Figure S2.** EdU assay using flow cytometry was applied for cell proliferation analysis in AGS and HGC-27 cells following overexpression or knockdown of RECK. **Figure S3.** Quantitative analysis of protein expression levels. **Figure S4.** CALD1 depletion rescued the inhibitory phenotype of GC cells by RECK overexpression. **Figure S5.** CALD1 upregulation inhibited the stimulatory phenotype of GC cells by RECK knockdown.

## Data Availability

The data supporting this study’s findings are available on request from the corresponding author.

## Abbreviations

GC: Gastric cancer
RECK: Reversion-inducing cysteine-rich protein with Kazal motifs
MMPs: Matrix metalloproteinases
CSCs: Cancer stem cells
CPTAC: The National Cancer Institute’s Clinical Proteomic Tumor Analysis Consortium
STAD: Stomach adenocarcinoma
COAD: Colon adenocarcinoma
ESCA: Esophageal carcinoma
READ: Rectum adenocarcinoma
TCGA: The Cancer Genome Atlas
GEO: The Gene Expression Omnibus
DEGs: Differentially Expressed Genes
GTEx: The Genotype-Tissue Expression
EMT: Epithelial-to-mesenchymal transition
GO: Gene ontology
KEGG: Kyoto Encyclopedia of Genes and Genomes
GSEA: Gene Set Enrichment Analysis
GSVA: Gene set variation analysis
TME: Tumor Microenvironment Estimation
RT-qPCR: Reverse transcription quantitative real-time polymerase chain reaction
CCK-8: The Cell Counting Kit
ROC: Receiver operating characteristic curve
AUC: Area under curve
OS: Overall survival
mDNAsi: DNA methylation-based stemness index
mRNAsi: mRNA expression-based stemness index
PCA: Principal component analysis
ssGSEA: Single sample Gene Set Enrichment Analysis
TBHQ: *tert*-Butylhydroquinone
PPI: Protein–protein interaction
CALD1: Caldesmon 1
TIMP2: TIMP metallopeptidase inhibitor 2
ADGRA2: Adhesion G protein-coupled receptor A2
STRING: The Search Tool for the Retrieval of Interacting Genes database
