# Supplementary material for: Identification of RECK as a protective prognostic indicator and a tumor suppressor through regulation of the ERK/MAPK signaling pathway in gastric cancer
Source: J Transl Med. 2023 Oct 30;21:766. doi: 10.1186/s12967-023-04644-z (PMC10614389; doi:10.1186/s12967-023-04644-z)
Supplement: Supplementary file 2 — Additional file 2: Figure S1. Time-dependent ROC curve analysis for survival prediction by RECK expression level in patients with STAD from GSE13861 and GSE28541 datasets. Figure S2. EdU assay using flow cytometry was applied for cell proliferation analysis in AGS and HGC-27 cells following overexpression or knockdown of RECK. Figure S3. Quantitative analysis of protein expression levels. Figure S4. CALD1 depletion rescued the inhibitory phenotype of GC cells by RECK overexpression. Figure S5. CALD1 upregulation inhibited the stimulatory phenotype of GC cells by RECK knockdown. [file 12967_2023_4644_MOESM2_ESM.docx]

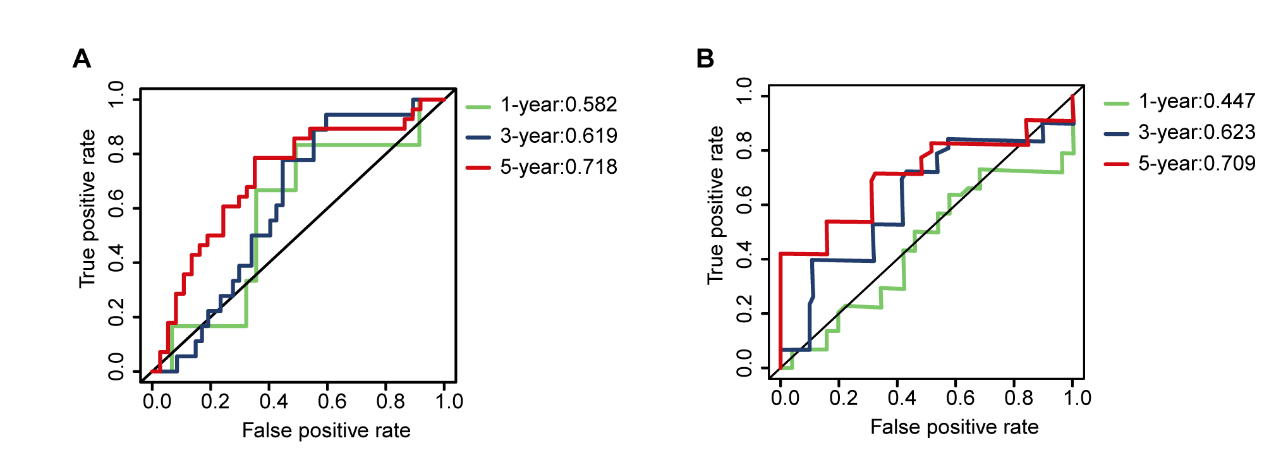


**Fig. S1** Time-dependent ROC curve analysis for survival prediction by RECK expression level in patients with STAD from GSE13861 (A) and GSE28541 (B) datasets.


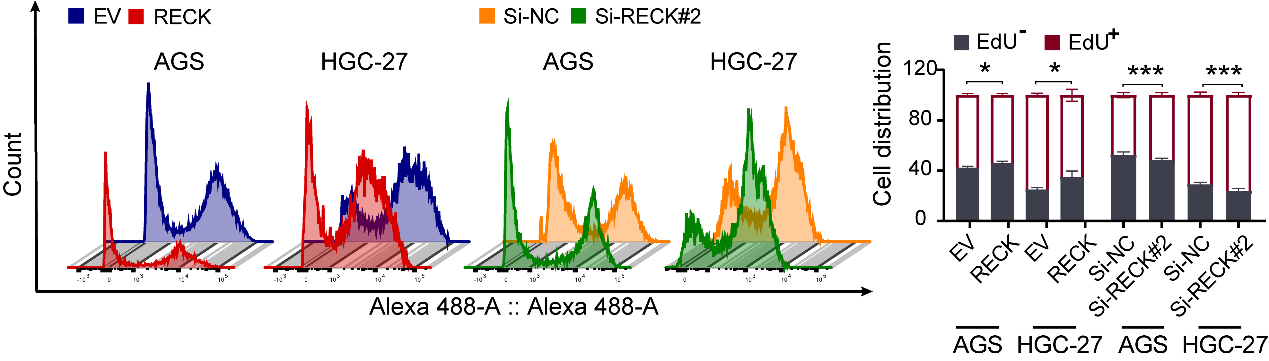


**Fig. S2** EdU assay using flow cytometry was applied for cell proliferation analysis in AGS and HGC-27 cells following overexpression or knockdown of RECK. (*p < 0.05, **p < 0.01, ***p < 0.001, ns = not significant)


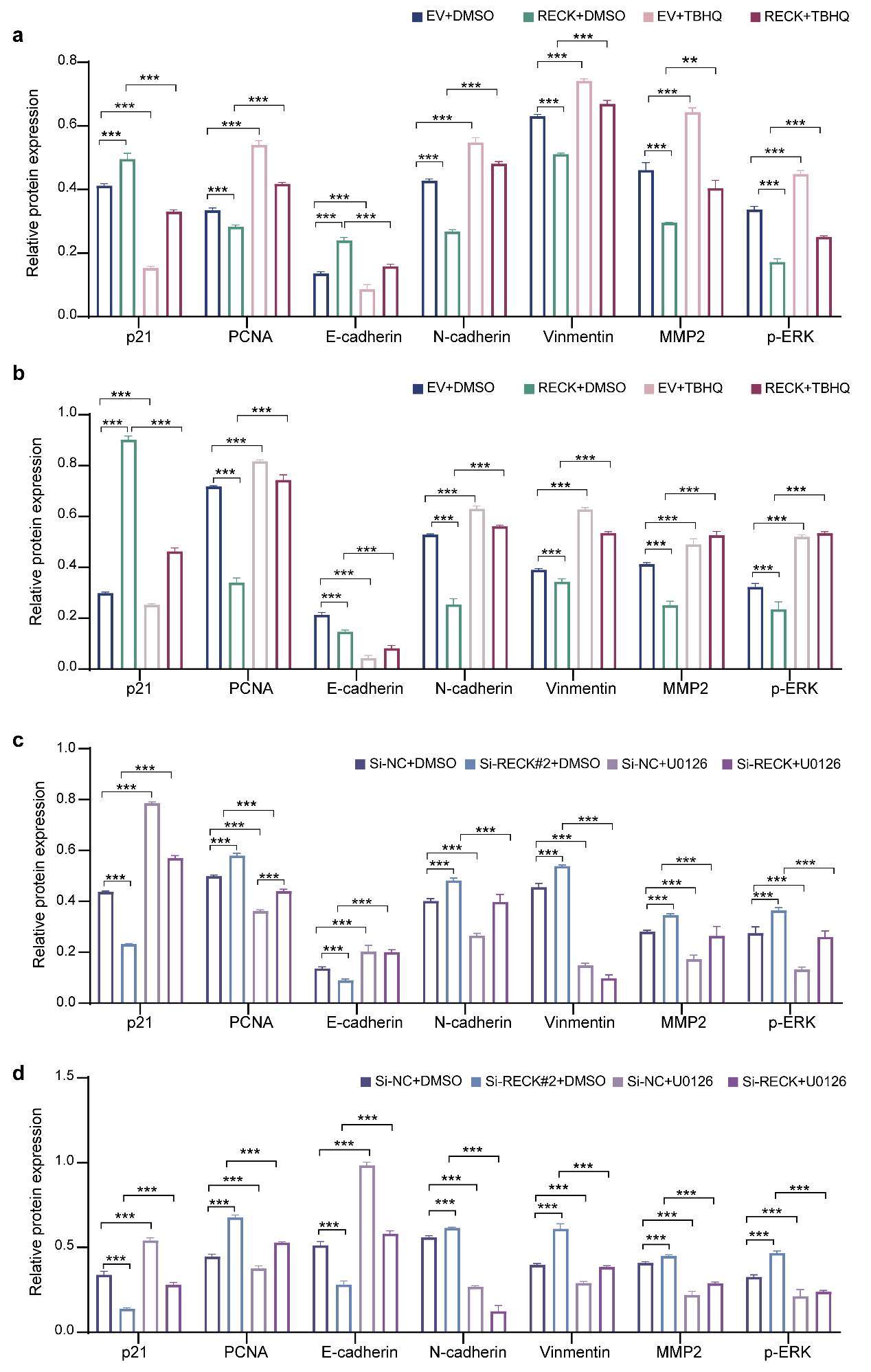


**Fig. S3** Quantitative analysis of protein expression levels.

**A, B** The quantified protein level of proliferation markers, EMT markers, and MAPK signaling markers in AGS (A) and HGC-27 (B) cells following overexpression of RECK and treatment with the ERK activator TBHQ.

**C, D** The quantified protein level of proliferation markers, EMT markers, and MAPK signaling markers in AGS (A) and HGC-27 (B) cells following knockdown of RECK and treatment with the ERK inhibitor PD98059. (*p < 0.05, **p < 0.01, ***p < 0.001, ns = not significant.)


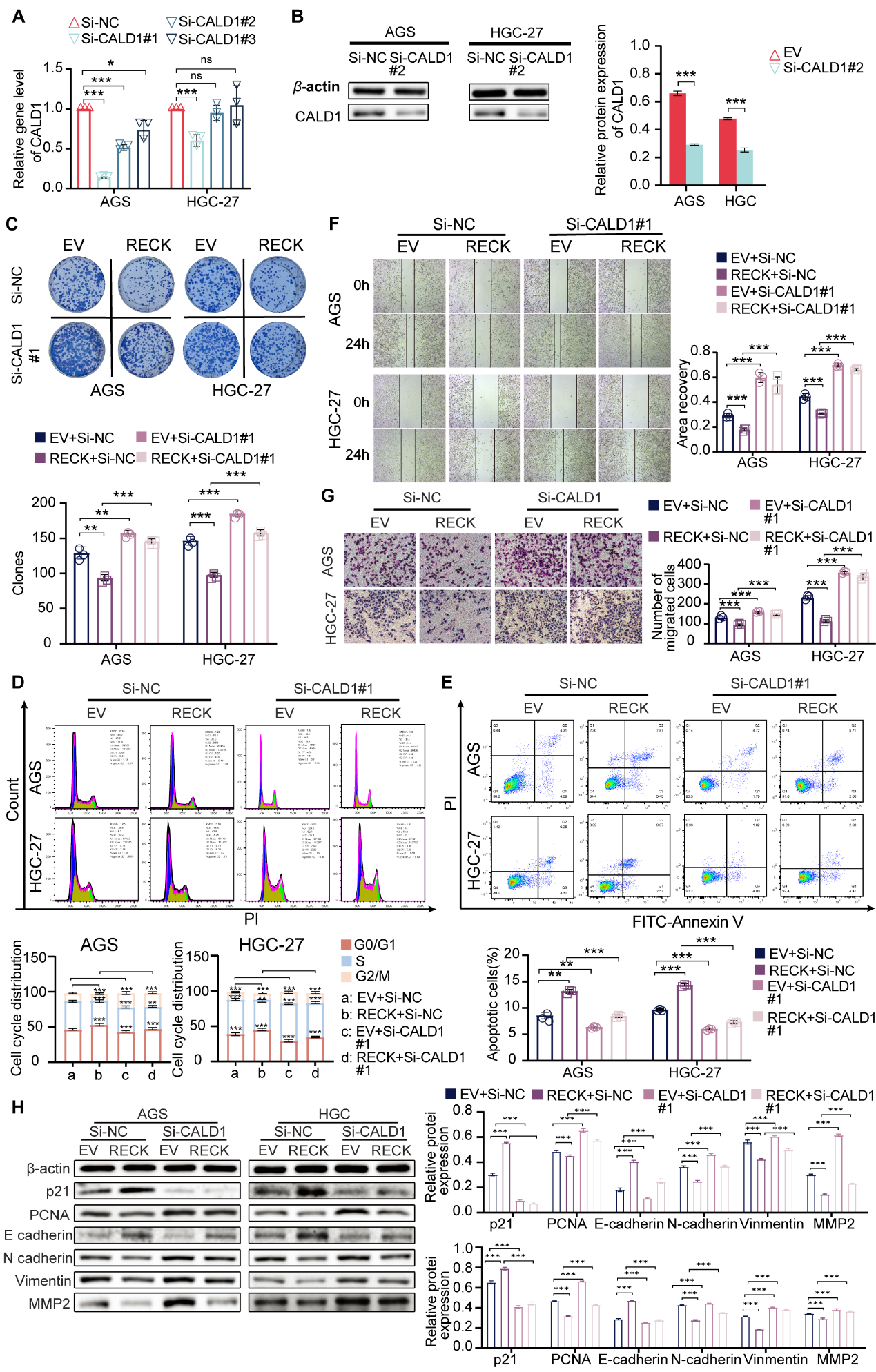


**Fig. S4.** CALD1 depletion rescued the inhibitory phenotype of GC cells by RECK overexpression. **A-B** The efficiency of CALD1 knockdown was detected by qRT-PCR (A) and western blot assays in AGS and HGC-27 cells (B). **C** Colony formation assay for the evaluation of GC cell proliferation in RECK-overexpressed AGS and HGC-27 cells with CALD1 depletion. **D-E** Cell cycle (D) and cell apoptosis (E) were detected by flow cytometry in RECK-overexpressed AGS and HGC-27 cells with CALD1 depletion. **F-G** The migration and invasion abilities of GC cells were measured using wound-healing and Matrigel invasion assays in RECK-overexpressed AGS and HGC-27 cells with CALD1 depletion. **H** Western blot representations of cell proliferation markers and EMT markers levels in RECK-overexpressed AGS and HGC-27 cells with CALD1 depletion. (*p < 0.05, **p < 0.01, ***p < 0.001, ns = not significant.)


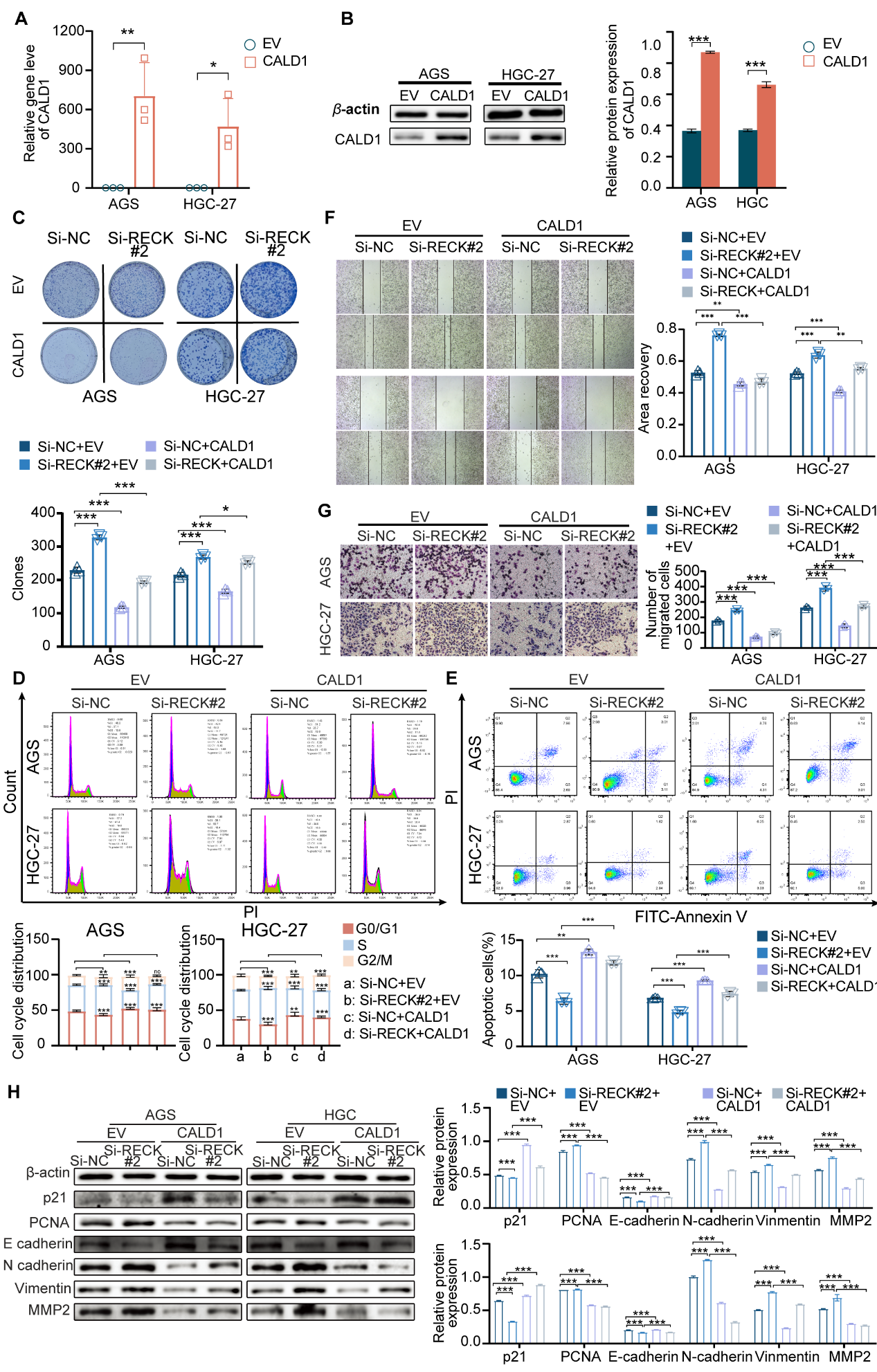


**Fig. S5.** CALD1 upregulation inhibited the stimulatory phenotype of GC cells by RECK knockdown. **A-B** The efficiency of CALD1 overexpression was detected by qRT-PCR (A) and western blot assays in AGS and HGC-27 cells (B). **C** Colony formation assay for the evaluation of GC cell proliferation in RECK-depleted AGS and HGC-27 cells with CALD1 overexpression. **D-E** Cell cycle (D) and cell apoptosis (E) were detected by flow cytometry in RECK-depleted AGS and HGC-27 cells with CALD1 overexpression. **F-G** The migration and invasion abilities of GC cells were measured using wound-healing and Matrigel invasion assays in RECK-depleted AGS and HGC-27 cells with CALD1 overexpression. **H** Western blot representations of cell proliferation markers and EMT markers levels in RECK- depleted AGS and HGC-27 cells with CALD1 overexpression. (*p < 0.05, **p < 0.01, ***p < 0.001, ns = not significant.)
